# Perinatal Factors Associated with Neonatal Microbiome Development and Their Implications for Nursing Practice: An Integrative Review

**DOI:** 10.3390/nursrep16090341

**Published:** 2026-09-18

**Authors:** Martina Feliu, Maria Gallo-Fernández, Adrián Bouzas, Maria Isabel Buceta-Toro, Clara Gomez-Urios

**Affiliations:** 1 Faculty of Nursing and Physiotherapy Salus Infirmorum, Pontifical University of Salamanca, 28015 Madrid, Spain; mfeliumo.sal@upsa.es (M.F.); mgallofe@upsa.es (M.G.-F.); abouzasmu@upsa.es (A.B.); 2 Nutrition and Food Science Area, Faculty of Pharmacy and Food Science, University of Valencia, Av. Vicent Andrés Estellés 22, 46100 Burjassot, Spain; clara.urios@uv.es

**Keywords:** neonatal microbiome, neonatal microbiota, microbial colonization, breastfeeding, mode of delivery, perinatal antibiotics, nursing care, newborn

## Abstract

**Background**: The neonatal microbiome plays a fundamental role in immune, metabolic, and physiological development from the earliest stages of life. Its establishment is influenced by several perinatal factors, many of which are closely related to nursing care practices. **Objective**: This study aimed to identify the principal perinatal factors associated with neonatal microbiome development and, secondarily, to explore their implications for nursing practice based on the available evidence. **Methods**: An integrative review was conducted using PubMed, Scopus, Web of Science, and CINAHL. Studies published between 2016 and 2026 involving newborns or neonates and addressing factors influencing neonatal microbiome development were considered. Following screening, eligibility assessment, and methodological quality appraisal, 14 studies were included in the final synthesis. **Results**: The evidence consistently identified mode of delivery, breastfeeding, and perinatal antibiotic exposure as the perinatal factors most consistently associated with neonatal microbial colonization. Vaginal delivery and breastfeeding were associated with greater abundances of beneficial bacterial genera, including *Bifidobacterium*, *Lactobacillus*, and *Bacteroides*, whereas cesarean delivery and antibiotic exposure were associated with reduced microbial diversity and altered colonization patterns. Emerging interventions such as vaginal microbial transfer showed promising results, although current evidence remains limited. Nursing-related practices, particularly breastfeeding support, promotion of early skin-to-skin contact, and participation in antimicrobial stewardship, were identified as key strategies for supporting healthy microbiome development. **Conclusions**: Current evidence suggests that nursing care may play an important role in promoting healthy neonatal microbial colonization through interventions that support physiological birth processes, breastfeeding, and appropriate antibiotic use. Further research is needed to evaluate the direct impact of specific nursing interventions on neonatal microbiome development and subsequent health outcomes.

## 1. Introduction

The neonatal period represents a critical window for the establishment of the human microbiome, a complex microbial ecosystem that plays an essential role in immune, metabolic, and physiological development from the earliest stages of life [1,2,3]. During the first days and weeks after birth, microbial colonization occurs rapidly and may influence intestinal maturation, immune tolerance, resistance to pathogens, and subsequent susceptibility to inflammatory, allergic, metabolic, and gastrointestinal disorders [3,4,5].

The composition of the neonatal microbiome is shaped by multiple maternal, perinatal, environmental, and healthcare-related factors. Among the most consistently identified determinants are mode of delivery, breastfeeding, maternal microbiota, skin-to-skin contact, exposure to antibiotics, hospitalization, and the neonatal care environment [6,7,8,9,10,11,12]. These factors are particularly relevant because the neonatal period is characterized by rapid microbial succession and high susceptibility to external influences.

Mode of delivery is one of the main determinants of early microbial colonization. Vaginal birth promotes exposure to maternal vaginal and intestinal microorganisms, including members of the genera *Lactobacillus*, *Bifidobacterium*, and *Bacteroides*, which have been associated with immune maturation and a more favorable pattern of intestinal colonization [8,13]. In contrast, infants born by cesarean section generally show delayed colonization by these taxa and greater exposure to skin- and hospital-associated microorganisms, including *Staphylococcus*, *Streptococcus*, *Clostridium*, and *Klebsiella* [10,14]. However, differences between elective and emergency cesarean delivery should be considered, as exposure to labor and ruptured membranes may partially modify the neonatal microbial profile [1,10,13,14].

Although cesarean delivery has been associated with altered microbial patterns during the first months of life, the long-term clinical significance of these differences remains uncertain. Associations have been reported between early dysbiosis and a greater risk of asthma, atopic dermatitis, food allergy, type 1 diabetes, and inflammatory bowel disease, but current evidence does not establish definitive causal relationships [10,13]. Therefore, mode of delivery should not be interpreted as an isolated determinant but rather as one component of a broader network of perinatal exposures.

Breastfeeding is another major modulator of neonatal microbial development. Human milk provides not only nutrients but also live microorganisms, human milk oligosaccharides, lactoferrin, lysozyme, and secretory immunoglobulin A, all of which contribute to intestinal maturation and selective growth of beneficial bacteria [15,16,17]. In particular, human milk oligosaccharides support the proliferation of *Bifidobacterium* species and the production of short-chain fatty acids, which participate in epithelial integrity, immune regulation, and protection against pathogenic colonization [15,16,17,18].

Evidence suggests that the effect of breastfeeding on the microbiome may follow a dose–response pattern. Exclusive breastfeeding has been associated with a greater predominance of beneficial microorganisms, whereas mixed feeding and formula feeding are generally linked to less favorable microbial profiles [15,17]. Nevertheless, these associations are influenced by several confounding factors, including maternal diet, antibiotic exposure, gestational age, mode of delivery, and duration of breastfeeding.

Perinatal antibiotic exposure is also associated with detectable alterations in the neonatal intestinal microbiota. Maternal intrapartum and neonatal antibiotic administration have been linked to reduced abundance of *Bifidobacterium* and increased colonization by opportunistic microorganisms such as *Klebsiella pneumoniae* [10,19,20]. Although antibiotics remain essential for the prevention and treatment of maternal and neonatal infections, their potential effect on microbial development reinforces the need for appropriate indication, administration, and antimicrobial stewardship.

Early skin-to-skin contact may also contribute to neonatal microbial acquisition by facilitating transfer of maternal skin microorganisms and supporting early breastfeeding initiation [8,10,11]. This practice may be particularly relevant after cesarean delivery, although it should not be considered a direct replacement for exposure occurring during vaginal birth. Its potential microbiological benefits coexist with well-established effects on neonatal thermoregulation, cardiorespiratory stability, bonding, and breastfeeding establishment [8,21,22].

The neonatal care environment is especially important in preterm infants and those admitted to neonatal intensive care units. Prematurity, prolonged hospitalization, invasive procedures, parenteral nutrition, and repeated antibiotic exposure are associated with reduced microbial diversity and increased abundance of hospital-associated microorganisms such as *Enterococcus*, *Klebsiella*, and *Staphylococcus* [5,10,18]. In these settings, infection prevention, vascular access care, promotion of human milk feeding, and reducing avoidable environmental exposures are particularly relevant nursing responsibilities [10,11,15,16].

**Figure 1:**
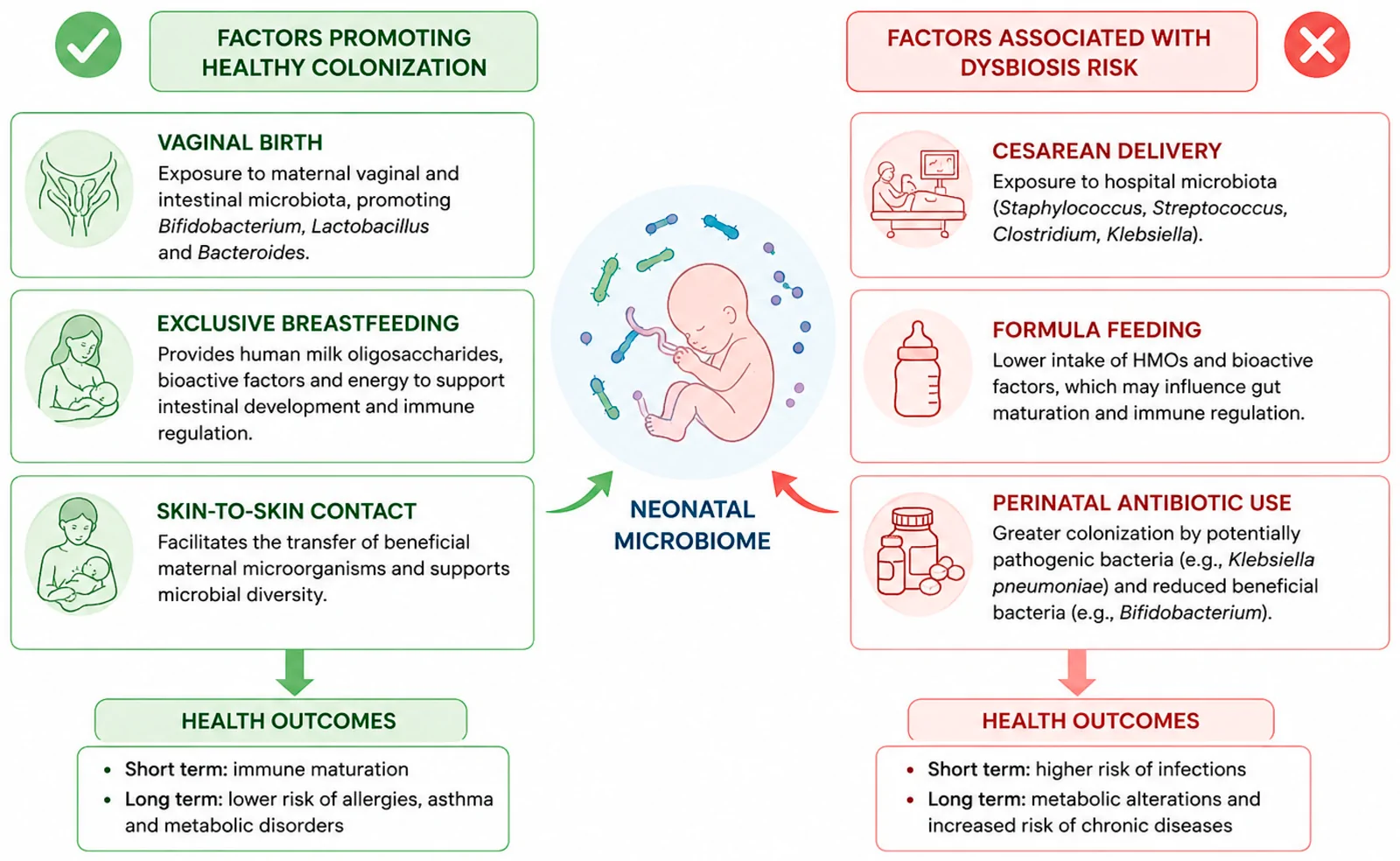
Determinants of perinatal colonization and their impact on health.

Nurses are directly involved in many of the practices that influence neonatal microbial development (Figure 1). Their role includes antenatal education, support for physiological and respectful birth, facilitation of immediate skin-to-skin contact, breastfeeding counselling, early identification of lactation difficulties, safe antibiotic administration, infection prevention, and family education [8,15,16,21,22]. However, most available studies have evaluated perinatal factors rather than nursing interventions as independent exposures. Consequently, the specific contribution of nursing care to neonatal microbial colonization remains insufficiently defined.

Despite growing evidence regarding the influence of perinatal factors on neonatal microbiome development, their implications for nursing practice remain insufficiently characterized. Nurses are directly involved in several aspects of perinatal care that may influence microbial colonization, including breastfeeding support, skin-to-skin contact, infection prevention, and antimicrobial stewardship [21,22].

Therefore, this review sought to address the following research questions: What perinatal factors are associated with neonatal microbiome development, and what are their implications for nursing practice? This integrative review aimed to identify the principal perinatal factors associated with neonatal microbial colonization and, secondarily, to explore how nursing care may support healthy microbiome development on the basis of the available evidence.

## 2. Materials and Methods

### 2.1. Review Design

This integrative review was conducted following the methodological framework proposed by Whittemore and Knafl, which allows the inclusion, critical appraisal, and synthesis of evidence from studies with diverse methodological designs to provide a comprehensive understanding of complex healthcare topics. This approach is particularly appropriate for emerging fields such as neonatal microbiome research, where evidence derives from observational studies, clinical trials, and other quantitative designs. The review process comprised five stages: problem identification, literature search, data evaluation, data analysis, and presentation of the findings [23]. To enhance transparency in the study selection process, the review was reported in accordance with the Preferred Reporting Items for Systematic Reviews and Meta-Analyses (PRISMA) [24] guidelines.

The review question was structured according to the Population–Exposure–Outcome (PEO) framework. The Population (P) comprised newborns and neonates; the Exposure (E) included perinatal factors potentially influencing microbial colonization, such as mode of delivery, breastfeeding, maternal factors, and perinatal antibiotic exposure; and the Outcome (O) was neonatal microbiome development. This framework was selected because the review primarily examined exposures and associated outcomes rather than the effectiveness of a specific clinical intervention.

### 2.2. Search Strategy

A literature search was conducted between December 2025 and March 2026 using four electronic databases: PubMed, Scopus, Web of Science, and CINAHL. Relevant studies were identified through a combination of Medical Subject Headings (MeSH), Health Sciences Descriptors (DeCS), and free-text keywords related to neonatal microbiome development, perinatal factors, and nursing care. Search terms were organized according to the PEO framework, combining terms related to the neonatal population, perinatal exposures, and neonatal microbiome outcomes.

Search terms were organized according to the PEO framework and combined using the Boolean operators AND and OR. The search strategy was adapted to the syntax and indexing requirements of each database. Searches were limited to studies published between 2016 and 2026, in English or Spanish, and review articles were excluded using the available database filters. The complete search strategies used in each database, together with the corresponding filters and limits, are provided in Supplementary Table S1 to improve transparency and reproducibility.

### 2.3. Eligibility Criteria

Studies were considered eligible for inclusion when they met the following criteria: (i) publication between 2016 and 2026; (ii) human participants; (iii) newborns or neonates aged up to one month; (iv) original research articles, including observational studies, clinical trials, and intervention studies; and (v) publication in English or Spanish.

Studies were excluded if they involved animal models, children older than one month, review articles, systematic reviews, editorials, conference proceedings, or publications written in languages other than English or Spanish.

### 2.4. Study Selection

The selection process was performed in several stages. First, all records retrieved from the electronic databases were screened according to the predefined eligibility criteria. Subsequently, titles and abstracts were reviewed to assess their relevance to the review question. Potentially eligible studies underwent full-text evaluation. Study selection, including title and abstract screening and full-text eligibility assessment, was conducted by one reviewer using the predefined eligibility criteria.

Duplicate records identified across databases were removed before full-text assessment. Studies meeting all eligibility criteria and demonstrating sufficient methodological quality were included in the final synthesis.

A total of 3608 records were initially identified across the four databases. Following application of the eligibility criteria, removal of duplicates, title and abstract screening, and full-text assessment, 16 studies were considered eligible for detailed evaluation. After methodological quality assessment, 14 studies were included in the final review.

### 2.5. Quality Assessment

The methodological quality of the included studies was assessed using a structured appraisal framework developed specifically for this integrative review. Given the methodological heterogeneity of the included evidence, which comprised prospective and longitudinal cohort studies, cross-sectional studies, randomized controlled trials, and non-randomized intervention studies, the use of a single design-specific appraisal checklist was considered difficult to apply consistently across all studies. Therefore, a common set of methodological domains was used to allow a consistent appraisal across the heterogeneous study designs included in the review.

The appraisal framework was informed by methodological domains commonly addressed in established critical appraisal approaches, including the Critical Appraisal Skills Programme (CASP) checklists and the Joanna Briggs Institute (JBI) Critical Appraisal Tools. However, it was not intended to constitute a validated alternative to these instruments. Rather, it was used as a review-specific framework to systematically examine methodological characteristics considered relevant to the present integrative review.

The assessment included eleven predefined criteria evaluating (i) clarity of the study objective; (ii) adequacy of neonatal population definition; (iii) appropriateness of the study design; (iv) description of the intervention or exposure; (v) relevance of microbiome-related outcomes; (vi) reporting of key perinatal factors such as mode of delivery, breastfeeding, and antibiotic exposure; (vii) transparency of data collection and analysis methods; (viii) consistency between objectives and reported results; (ix) adequacy of the conclusions based on the reported findings; (x) relevance to the review question; and (xi) overall methodological quality.

Each criterion was scored on a three-point scale (0 = criterion not fulfilled, 1 = partially fulfilled, and 2 = fully fulfilled), yielding a maximum possible score of 22 points. Studies scoring 18–22 points were classified as high quality, those scoring 12–17 points as moderate quality, and those scoring 0–11 points as low quality. These scoring thresholds were established by the authors for this review and have not been externally validated. To improve the methodological robustness of the review, studies classified as low quality were excluded from the final evidence synthesis. Methodological quality scores and classifications are presented in Table 1 and Supplementary Tables S2 and S3.

Because this review-specific framework has not undergone formal validation, the resulting scores and quality categories should be interpreted as an aid to the structured appraisal and comparison of the included studies rather than as validated measures of methodological quality. This limitation was considered when interpreting the findings of the review.

### 2.6. Data Extraction and Synthesis

Data were extracted from each included study using a standardized template. Extracted information included authorship, year of publication, study design, sample size, population characteristics, gestational age when reported, clinical setting and neonatal intensive care unit (NICU) status, exposure or intervention, biological specimen analyzed, sampling time, microbiological or sequencing methods, bioinformatics or analytical approaches, microbiome-related outcome measures, potential confounders or adjustment variables, effect estimates when reported, and principal findings relevant to the review question. General study characteristics are presented in Table 2, while detailed microbiological and analytical characteristics are provided in Supplementary Table S4. Information not reported in the original studies is indicated as “NR” (not reported).

## 3. Results and Discussion

### 3.1. Study Selection, Characteristics, and Methodological Quality

The literature search identified a total of 3608 records across four electronic databases: Scopus (*n* = 2683), Web of Science (*n* = 514), PubMed (*n* = 113), and CINAHL (*n* = 298). After applying the predefined database filters, 3317 records were excluded, leaving 291 records for title and abstract screening. Of these, 245 records were excluded because they were not directly relevant to the review question, leaving 46 potentially eligible records for further assessment.

**Figure 2:**
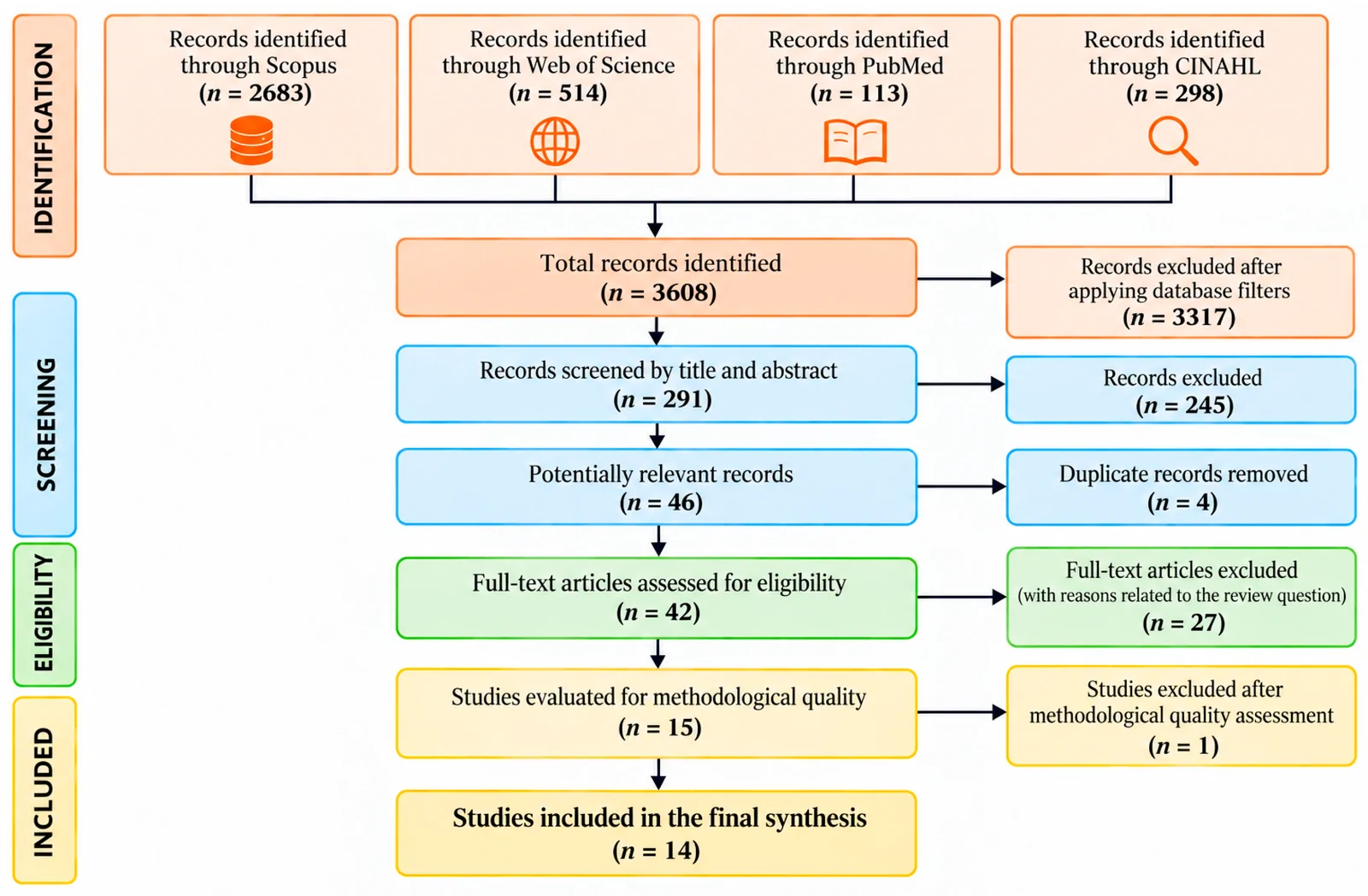
PRISMA flow diagram of the study selection process.

A total of 46 potentially relevant studies were retained for further assessment. After duplicate removal (*n* = 4), 42 full-text articles were evaluated for eligibility. Following full-text assessment, 26 articles were excluded because they did not meet the predefined eligibility criteria regarding study design and publication type, as they corresponded to reviews or other non-original research publications. The remaining 16 studies underwent methodological quality assessment. Of these, two studies were classified as low quality and excluded, resulting in 14 studies being included in the final evidence synthesis (Figure 2).

The included studies comprised a range of methodological designs, including prospective and longitudinal cohort studies, cross-sectional studies, comparative observational studies, one randomized controlled trial, and one non-randomized intervention study. Most investigations focused on factors influencing neonatal microbiome development, including mode of delivery, breastfeeding, perinatal antibiotic exposure, maternal characteristics, and interventions aimed at modifying microbial colonization patterns. A summary of the characteristics and principal findings of the included studies is presented in Table 2.

The studies were conducted across multiple geographic regions, including Europe, North America, and Asia, providing a broad perspective on neonatal microbiome development across diverse populations. Publication dates ranged from 2016 to 2025, with the majority of studies published during the last five years, reflecting the growing scientific interest in this field.

Sample sizes varied considerably, ranging from 18 mother–infant pairs to 120 mother–infant pairs. Most studies evaluated microbial colonization during the first days or weeks of life, although several included follow-up periods extending into infancy, allowing the assessment of longer-term microbiome development.

Methodological quality assessment identified ten studies as high quality, four as moderate quality, and two as low quality. High-quality studies generally demonstrated clearly defined objectives, appropriate study designs, comprehensive methodological descriptions, and outcomes directly related to neonatal microbiome development. Moderate-quality studies provided relevant evidence but showed certain methodological limitations. The two low-quality studies were excluded from the final evidence synthesis. Detailed quality scores and classifications are presented in Table 1 and Table S1.

### 3.2. Mode of Delivery and Neonatal Microbiome Development

Mode of delivery emerged as one of the perinatal factors most consistently associated with neonatal microbiome establishment. Across the studies included in this review, vaginal delivery was associated with microbial profiles characterized by higher abundances of taxa such as *Bifidobacterium*, *Lactobacillus*, and *Bacteroides*, whereas cesarean delivery was associated with lower microbial diversity, delayed microbial maturation, and distinct colonization patterns [23,28,34].

Akagawa et al. [23] reported that infants born vaginally and exclusively breastfed exhibited higher abundances of *Bifidobacterium* and *Lactobacillus*, while cesarean-delivered infants receiving formula feeding showed increased levels of *Clostridium* and lower overall microbial diversity. Similarly, Matharu et al. [29] demonstrated that *Bacteroides* colonization was markedly reduced in cesarean-born infants during the first days of life, with only partial recovery observed during subsequent weeks and months. These findings suggest that microbial exposure associated with vaginal birth may contribute to differences in early neonatal microbiome establishment.

The observed differences are biologically plausible, as vaginally delivered infants are exposed to maternal vaginal and intestinal microbiota during birth, facilitating the vertical transmission of microorganisms involved in immune and metabolic development. In contrast, cesarean delivery limits this exposure and increases contact with environmental and hospital-associated microorganisms, resulting in a distinct colonization pattern during the early postnatal period [34].

Several studies have proposed that alterations in microbial colonization associated with cesarean delivery may contribute to immune dysregulation and increased susceptibility to certain health conditions later in life. Although the causal relationship remains incompletely understood, the consistent reduction in beneficial microbial taxa observed across studies highlights the importance of strategies aimed at supporting microbial establishment in cesarean-delivered infants [23,28].

Evidence from intervention studies further supports the importance of early microbial exposure. Domínguez-Bello et al. [35] demonstrated that vaginal microbial transfer following cesarean birth partially restored neonatal microbial communities, producing bacterial profiles more closely resembling those observed in vaginally delivered infants during the first weeks of life. While these findings are promising, the available evidence remains limited, and concerns regarding safety and standardization continue to restrict the routine implementation of this practice.

From a nursing perspective, these findings reinforce the importance of promoting physiological birth whenever clinically appropriate and implementing evidence-based practices that may mitigate microbial alterations associated with cesarean delivery. Early skin-to-skin contact, avoidance of unnecessary mother–infant separation, and support for early breastfeeding initiation represent practical interventions that may contribute to a more favorable microbial environment during this critical developmental period [21,22].

### 3.3. Breastfeeding and Early Microbial Colonization

Breastfeeding was consistently associated with differences in early microbial colonization. Across the included studies, breastfed infants generally showed higher abundances of taxa commonly associated with the breastfed infant microbiome, particularly *Bifidobacterium*, compared with formula-fed infants [30,36,37].

Raspini et al. [30] reported that microbial colonization patterns established during the first months of life were strongly influenced by feeding practices, with breastfed infants showing significantly higher levels of *Bifidobacterium* at six months of age than formula-fed infants. These findings suggest that the effects of breastfeeding extend beyond the neonatal period and may contribute to the long-term maturation of the intestinal microbiota.

The observed associations between breastfeeding and neonatal microbiome composition are biologically plausible given the characteristics of human milk. Human milk provides not only essential nutrients but also a wide range of bioactive compounds, including human milk oligosaccharides, immunoglobulins, antimicrobial peptides, and beneficial microorganisms that contribute to intestinal colonization and immune development. These components selectively promote the growth of bacterial taxa associated with gut health while limiting the proliferation of potentially pathogenic microorganisms [15,16,17].

In addition to feeding practices, maternal nutrition emerged as an important factor influencing neonatal microbial development. Ajeeb et al. [36] observed that mothers with higher-quality dietary patterns exhibited greater microbial diversity in breast milk, suggesting that maternal nutrition may indirectly influence neonatal colonization through modifications in milk microbiota composition. Similarly, Cabrera-Rubio et al. [37] demonstrated associations between maternal dietary quality during pregnancy and differences in the abundance of *Bifidobacterium* and *Bacteroides* in infants during the first month of life.

These findings indicate that the benefits of breastfeeding cannot be considered independently of maternal health and nutrition. Rather, neonatal microbial development appears to result from a complex interaction between maternal diet, breast milk composition, and infant feeding practices. Although further research is required to clarify the underlying mechanisms, the available evidence supports the existence of a maternal–milk–infant microbial continuum that may influence neonatal health outcomes [36,37].

From a nursing perspective, breastfeeding support represents a relevant component of routine perinatal care that may also be associated with neonatal microbial development. Nurses play a central role in facilitating breastfeeding initiation, supporting its continuation, identifying early lactation difficulties, and providing education to mothers throughout the postpartum period. Furthermore, the findings of this review suggest that nutritional counselling during pregnancy and lactation may represent an additional opportunity to support neonatal microbiome development, extending the role of nursing beyond breastfeeding promotion alone [15,16,17,25,36,37].

### 3.4. Impact of Perinatal Antibiotic Exposure on Neonatal Microbiome Development

Perinatal antibiotic exposure emerged as another factor consistently associated with neonatal microbial colonization. Although antibiotic administration is frequently necessary for the prevention and treatment of maternal and neonatal infections, the studies reviewed consistently reported associations between antibiotic exposure and alterations in the early establishment of the neonatal microbiome [33].

The evidence indicated that infants exposed to antibiotics during the perinatal period exhibited lower abundances of beneficial bacterial genera, particularly *Bifidobacterium*, together with increased colonization by opportunistic microorganisms such as *Klebsiella pneumoniae* [33]. These alterations were observed not only following direct neonatal antibiotic administration but also after maternal antibiotic exposure during pregnancy or delivery, suggesting that antimicrobial therapy may interfere with vertical microbial transmission between mother and infant.

Zhou et al. [33] demonstrated that maternal, neonatal, and combined antibiotic exposure were all associated with measurable changes in microbial composition. Notably, maternal antibiotic administration alone was sufficient to modify neonatal microbial colonization patterns, highlighting the importance of considering indirect effects of antimicrobial therapy on neonatal microbiome development. The authors also reported an association between prenatal antibiotic exposure and an increased risk of neonatal sepsis, further emphasizing the complexity of balancing infection prevention with microbiome preservation.

These findings support growing concerns regarding the potential consequences of excessive or unnecessary antibiotic use during the perinatal period. While antimicrobial therapy remains essential in many clinical situations, the evidence suggests that careful assessment of indications, duration, and timing may be important for minimizing unintended effects on neonatal microbial colonization.

Nursing professionals play a key role in antimicrobial stewardship programs through the safe administration of prescribed treatments, monitoring of adverse effects, patient education, and participation in infection prevention strategies. Furthermore, nurses are uniquely positioned to promote compensatory practices that may support microbial recovery following antibiotic exposure, particularly breastfeeding and early mother–infant contact, both of which have been associated with more favorable microbial development [21,22,33].

Although current evidence supports an association between antibiotic exposure and microbiome alterations, most available studies are observational in nature. Therefore, further longitudinal research is required to determine the long-term clinical implications of these microbial changes and to identify strategies capable of mitigating their potential impact on infant health [33].

### 3.5. Implications for Nursing Practice

The findings of this integrative review suggest that several areas of routine nursing practice are relevant to perinatal exposures associated with neonatal microbiome development. However, most included studies evaluated perinatal factors rather than nursing-led interventions directly. Therefore, the nursing implications derived from this review should be interpreted as clinically relevant considerations rather than evidence of a direct effect of nursing care on neonatal microbiome composition.

Breastfeeding support represents one of the most relevant nursing responsibilities identified in this review. Nurses play a central role in facilitating early breastfeeding initiation, providing lactation counselling, identifying and managing breastfeeding difficulties, and encouraging exclusive breastfeeding whenever possible. These interventions are supported by consistent evidence demonstrating that breastfed infants develop more favorable microbial profiles characterized by a higher abundance of beneficial bacteria, particularly *Bifidobacterium* [15,16,17,30,37,38].

Similarly, nursing care contributes substantially to preserving physiological microbial transmission immediately after birth. Promoting uninterrupted skin-to-skin contact, minimizing unnecessary mother–infant separation, and encouraging rooming-in practices may facilitate the transfer of maternal microorganisms while simultaneously improving breastfeeding success, neonatal thermoregulation, cardiorespiratory stability, and parent–infant bonding [21,22]. These practices are particularly relevant following cesarean delivery, where opportunities for maternal microbial exposure are inherently reduced.

The review also highlights the importance of antimicrobial stewardship within neonatal and perinatal care. Although decisions regarding antibiotic prescription are made by physicians, nurses play a critical role in ensuring the appropriate administration of antimicrobial therapy, monitoring treatment-related adverse effects, promoting adherence to infection prevention protocols, and educating families about the rationale for antibiotic use. By participating in multidisciplinary antimicrobial stewardship programs, nurses may contribute to minimizing unnecessary antibiotic exposure and its potential impact on neonatal microbiome development [19,34].

Beyond direct clinical care, nursing professionals also have an essential educational role. Prenatal counseling regarding breastfeeding, healthy maternal nutrition, birth preparation, and early newborn care may improve parental understanding of the factors influencing neonatal microbial colonization. Furthermore, educating families about practices that support physiological microbiome development may facilitate informed decision-making and strengthen family-centered care.

Overall, several routine nursing practices, including breastfeeding support, facilitation of skin-to-skin contact, infection prevention, and participation in antimicrobial stewardship, are relevant to perinatal factors associated with neonatal microbial colonization. However, the direct effect of nursing-led interventions on neonatal microbiome composition remains insufficiently investigated. Future prospective and interventional studies are needed to evaluate these relationships directly.

## 4. Strengths and Limitations

This review provides an updated overview of the current evidence on perinatal factors influencing neonatal microbiome development and discusses their relevance for nursing practice. A major strength is the inclusion of studies with different methodological designs, which offers a broad perspective on a topic where evidence is still emerging. In addition, the literature search was performed across four electronic databases, and study selection followed the PRISMA 2020 [24] recommendations. Although a quantitative synthesis was not feasible, the narrative approach allowed the integration of findings from heterogeneous studies and the identification of consistent patterns across the available evidence.

Several limitations should also be considered. Most of the included studies were observational, which limits the possibility of establishing causal relationships between perinatal factors and neonatal microbiome development. Differences in study design, population characteristics, timing of sample collection, sequencing methods, and reported outcomes also introduced considerable heterogeneity, making a meta-analysis inappropriate. Furthermore, although this review focuses on implications for nursing practice, relatively few studies evaluated nursing interventions directly. Consequently, some of the proposed nursing implications are based on the interpretation of evidence regarding perinatal factors rather than on studies specifically designed to assess nursing-led interventions. Finally, the methodological quality assessment was conducted using a review-specific appraisal tool developed to allow consistent evaluation of the different study designs included in this review. While this approach helps comparison across heterogeneous studies, it has not been formally validated and should therefore be interpreted with this limitation in mind.

In addition, study selection was conducted by a single reviewer rather than independently by two reviewers. This represents an important methodological limitation, as the absence of independent duplicate screening may have increased the risk of erroneous exclusions and selection bias. Consequently, the study selection process and the resulting evidence synthesis should be interpreted with appropriate caution.

Finally, methodological quality was assessed using a review-specific appraisal framework designed to enable a consistent evaluation across the heterogeneous study designs included in this integrative review. Although the framework was informed by methodological domains considered in established critical appraisal approaches, it has not been formally validated. Consequently, the resulting quality scores and classifications should be interpreted with caution and should not be considered equivalent to assessments obtained using validated design-specific critical appraisal instruments.

## 5. Conclusions

This review identified mode of delivery, breastfeeding, and perinatal antibiotic exposure as the factors most consistently associated with early neonatal microbial colonization. Vaginal delivery and breastfeeding were generally associated with distinct microbial profiles characterized by higher abundances of taxa such as *Bifidobacterium*, *Lactobacillus*, and *Bacteroides*, whereas cesarean delivery and perinatal antibiotic exposure were associated with differences in microbial diversity and colonization patterns.

Several areas of nursing practice, including breastfeeding support, facilitation of early skin-to-skin contact, infection prevention, and participation in antimicrobial stewardship, are relevant to these perinatal exposures. However, because most available studies did not directly evaluate nursing-led interventions, no causal effect of nursing care on neonatal microbiome development can currently be established.

Emerging strategies such as vaginal microbial transfer have shown changes in microbial composition in cesarean-delivered infants, but current evidence remains insufficient to support their routine clinical implementation. Further prospective and interventional studies are needed to determine the direct effects of specific nursing and perinatal care practices on neonatal microbiome development and subsequent health outcomes.

## Figures and Tables

**Table 1:** Methodological quality of the articles.

Author	Year	Study Design	Quality Score	Quality Level
Akagawa et al. [23]	2019	Prospective cohort	22/22	High
Omaña-Covarrubias et al. [25]	2025	Correlational cohort	19/22	High
Farooq et al. [26]	2025	Longitudinal metagenomic study	17/22	Moderate
Seifert et al. [27]	2025	Observational microbiological study	14/22	Moderate
Mueller et al. [28]	2023	Randomized controlled trial	22/22	High
Matharu et al. [29]	2022	Longitudinal cohort	22/22	High
Raspini et al. [30]	2021	Prospective longitudinal cohort	22/22	High
Wu et al. [31]	2021	Comparative sequencing-based study	15/22	Moderate
García-Mantrana et al. [32]	2020	Mother–infant cohort	19/22	High
Zhou et al. [33]	2020	Comparative observational study	21/22	High
Dunn et al. [8]	2018	Analytical observational study	11/22	Low
Nagpal et al. [34]	2017	Longitudinal observational study	20/22	High
Dominguez-Bello et al. [35]	2016	Non-randomized intervention study	19/22	High
Ajeeb et al. [36]	2024	Cross-sectional study	18/22	High
Cabrera-Rubio et al. [37]	2024	Cross-sectional mother–infant cohort	18/22	High
Klymiuk et al. [38]	2021	Descriptive microbiological study	11/22	Low

**Table 2:** Characteristics and main findings of the studies included in the integrative review.

Author	Study Design	Sample Size	Population/Gestational Age	Clinical Setting/NICU Status	Exposure or Intervention	Main Findings
Akagawa et al. [23]	Prospective observational cohort	36 neonates	Healthy, normal-birth-weight, full-term Japanese neonates; GA approximately 37–42 weeks	Izumiotsu Municipal Hospital, Japan; NICU-admitted infants were excluded	Mode of delivery and feeding type	At 4 days, vaginally delivered infants showed greater bacterial diversity than cesarean-born infants. Differences between delivery groups were substantially reduced by 1 month; breastfeeding may contribute to this convergence.
Omaña-Covarrubias et al. [25]	Correlational cohort study	114 pregnant women	Women ≥ 18 years recruited at ≤12 weeks of gestation; maternal and neonatal microbiota evaluated	ISSSTE General Hospital, Pachuca, Mexico; NICU status NR	Maternal gut microbiota, particularly Firmicutes/Bacteroidetes ratio	Maternal microbiota composition was correlated with fetal growth parameters and neonatal microbiota, particularly for Firmicutes and Bacteroidetes.
Farooq et al. [26]	Longitudinal metagenomic study	18 mother–infant pairs; 125 fecal metagenomes	Mother–infant pairs followed from birth through 12 months; gestational characteristics varied in the source datasets	Publicly available longitudinal datasets; NICU status NR	Maternal–infant transmission of microbiota and antimicrobial-resistance genes	Multiple ARGs and associated microbial taxa were shared between mothers and infants over time, supporting persistent maternal–infant transmission of microbiome and resistome components.
Seifert et al. [27]	Observational microbiological study	39 newborn skin samples; 38 oral samples	Newborns of healthy-weight, overweight, and obese pregnant participants; GA NR	Hospital-based birth cohort, USA; NICU status NR	Maternal pre-pregnancy BMI and mode of delivery	Maternal obesity and delivery mode were associated with differences in newborn skin and oral microbiota. *Peptoniphilus* was more abundant in newborn skin of mothers with obesity, while *Ureaplasma* was more abundant orally after cesarean delivery.
Mueller et al. [28]	Double-blind randomized placebo-controlled trial	20 mother–infant dyads	Elective prelabor cesarean-born neonates	Inova Women’s/Children’s Hospital, USA; NICU status NR	Vaginal seeding versus sterile-saline placebo	Vaginal seeding increased maternal-to-neonate microbial transmission and altered skin and stool community composition, including reduced alpha diversity relative to placebo.
Matharu et al. [29]	Longitudinal cohort study	78 term infants; 280 infant samples	Healthy infants born at 37–42 weeks; birth weight > 2.5 kg	Finnish HELMi birth cohort; NICU status NR	Mode of delivery and associated perinatal exposures	Infant microbiota followed distinct developmental trajectories strongly associated with delivery mode. Low *Bacteroides* abundance characterized cesarean-associated trajectories and was also identified in a subgroup of vaginally delivered infants.
Raspini et al. [30]	Prospective longitudinal cohort	61 at birth; 53 at 6 months	Mother–infant pairs followed from birth; GA NR	Neonatal Unit, Fondazione IRCCS Policlinico San Matteo, Italy; NICU affiliation reported, individual NICU admission NR	Maternal BMI/gestational weight gain, delivery, feeding, weaning, siblings	Microbiota development at 6 months was associated with multiple interacting maternal and neonatal factors. Exclusive breastfeeding was associated with lower Shannon diversity, whereas non-exclusive breastfeeding was associated with higher *Ruminococcaceae* and *Flavonifractor* abundance.
Wu et al. [31]	Comparative sequencing-based study	25 mother–neonate pairs	Healthy full-term pregnancies, >37 weeks; all delivered by cesarean section	Gansu Provincial Maternity and Child-care Hospital, China; NICU status NR	Maternal microbial transmission	Shared OTUs and similarities in microbial community structure were found across maternal oral, amniotic-fluid, placental, and neonatal oral samples, indicating an association between maternal and neonatal oral microbiota.
García-Mantrana et al. [32]	Prospective mother–infant cohort	116 enrolled; 86 mother–neonate pairs included in paired microbiota analyses	Healthy mother–infant pairs from the MAMI birth cohort; GA NR	Hospital La Fe and Hospital Clínico Universitario de Valencia, Spain; NICU status NR	Maternal diet and maternal gut microbiota	Maternal microbial clusters were associated with dietary patterns and with neonatal microbiota. Higher fiber, omega-3 and polyphenol intake characterized one maternal microbial cluster, and associations with infant growth differed according to birth mode.
Zhou et al. [33]	Comparative observational study	98 mother–newborn pairs	Full-term and preterm births, with or without perinatal antibiotic exposure	Jinan University-affiliated Shenzhen Baoan Women’s and Children’s Hospital, China; NICU status NR	Perinatal antibiotic exposure and gestational age	Antibiotic exposure was associated with reduced maternal vaginal *Lactobacillus* abundance and altered maternal–neonatal microbial similarity. Gestational age was also associated with neonatal meconium *Lactobacillus* abundance, and dysbiosis was observed in newborns with early-onset sepsis.
Nagpal et al. [34]	Longitudinal observational study	89 infants	Healthy full-term Japanese infants	Gonohashi Obstetrics and Gynecology Hospital, Tokyo; NICU status NR	Delivery mode and feeding type	Cesarean-born infants showed greater carriage of toxigenic *C. perfringens* and lower levels of *B. fragilis* group, bifidobacteria, and lactobacilli during the first 6 months. Breastfeeding was associated with somewhat lower *C. perfringens* carriage.
Dominguez-Bello et al. [35]	Non-randomized pilot intervention study	18 mother–infant dyads: 7 vaginal, 7 unexposed cesarean, 4 cesarean + vaginal transfer	Term infants; cesarean and vaginal births	Hospital-based cohort; NICU status NR	Maternal vaginal microbial transfer after cesarean birth	Cesarean-born infants exposed to maternal vaginal fluids showed partial restoration of microbial communities, with anal, oral, and skin microbiota becoming more similar to those of vaginally delivered infants during the first month.
Ajeeb et al. [36]	Cross-sectional study	64 mother–infant dyads	Breastfed infants assessed during early (6–46 days) or late (109–184 days) lactation	Eight rural Mam-Mayan communities, Guatemala; NICU not applicable/NR	Maternal diet and human-milk microbiome	Maternal macro- and micronutrient intake was associated with human-milk microbial composition, and associations between specific milk taxa and infant growth differed according to lactation stage.
Cabrera-Rubio et al. [37]	Cross-sectional mother–infant cohort	120 mother–infant dyads	Infants from the Mediterranean MAMI birth cohort evaluated at 1 month	Spanish Mediterranean MAMI cohort; NICU status NR	Maternal diet during pregnancy	Higher maternal diet index scores were associated with differences in infant gut microbiota at 1 month, including microbial composition and diversity patterns, supporting an association between prenatal diet and early infant microbiota.

## Data Availability

No new data were created or analyzed in this study. Data sharing is not applicable to this article.

## Supplementary Materials

The following supporting information can be downloaded at: https://www.mdpi.com/article/10.3390/nursrep16090341/s1, Table S1. Complete search strategies used in the databases included in the integrative review; Table S2. Quality assessment; Table S3. Methodological quality; Table S4. Microbiome assessment and analytical characteristics of the included studies.

## Public Involvement Statement

No public involvement in any aspect of this research.

## Guidelines and Standards Statement

This manuscript was drafted against the PRISMA 2020 statement for reporting systematic reviews.

## Use of Artificial Intelligence

Artificial intelligence (AI) tools were used exclusively to support the preparation of the manuscript. Grammarly was used to improve the linguistic quality of the text, including grammar, spelling, readability, and overall cohesion. In addition, ChatGPT (OpenAI, GPT-4o) was used to assist in refining the visual presentation of the figures by improving their layout and graphical clarity. No AI tool was used for text generation, analysis, interpretation of results, or the formulation of conclusions. All scientific content, critical evaluation, and final decisions regarding the manuscript were performed and verified by the authors, who take full responsibility for the accuracy and integrity of the work.
