# Supplementary material for: Perinatal Factors Associated with Neonatal Microbiome Development and Their Implications for Nursing Practice: An Integrative Review
Source: Nurs Rep. 2026 Sep 18;16(9):341. doi: 10.3390/nursrep16090341 (PMC13610650; doi:10.3390/nursrep16090341)
Supplement: Supplementary file 1 [file nursrep-16-00341-s001.zip › nursrep-4494171-supplementary.pdf]

Perinatal Factors Associated with Neonatal Microbiome Development and Their Implications for Nursing Practice: An Integrative Review

Martina Feliu[1], Maria Gallo-Fernández[1], Adrian Bouzas[1], Maria Isabel Buceta-Toro[1*] ,Clara Gomez-Urios[1,2]

1 Faculty of Nursing and Physiotherapy Salus Infirmorum, Pontifical University of Salamanca, Madrid, 28015, Spain

2 Nutrition and Food Science Area. Faculty of Pharmacy and Food Science, University of Valencia. Av. Vicent Andrés Estellés 22, 46100, Burjassot, Spain

* Correspondence: mibucetato@upsa.es

SUPPLEMENTARY MATERIAL

Table S1. Complete search strategies used in the databases included in the integrative review.

Database	Complete search strategy	Filters
**PubMed**	(("newborn"[All Fields] OR "neonate"[All Fields]) AND ("Neonatal microbiome"[All Fields] OR "microbial colonization"[All Fields] OR "early Microbial colonization"[All Fields] OR "dysbiosis"[All Fields] OR "neonatal microbiota"[All Fields])) OR (("determinants of neonatal microbiota"[All Fields] OR "mode of delivery"[All Fields] OR "perinatal factors"[All Fields] OR "hospital environment"[All Fields]) AND ("Breastfeeding"[All Fields] OR "exclusive breastfeeding"[All Fields] OR "Breast milk microbiota"[All Fields] OR "skin-to-skin contact"[All Fields] OR "Kangaroo care"[All Fields] OR "early skin contact"[All Fields] OR "immediate postpartum contact"[All Fields]) AND ("nursing interventions"[All Fields] OR "neonatal nursing care"[All Fields] OR "evidence-based nursing"[All Fields] OR "nursing"[All Fields] OR "nurse"[All Fields]))	2016–2026; English or Spanish; reviews excluded
**Scopus**	TITLE-ABS-KEY(("newborn" OR "neonate") AND ("Neonatal microbiome" OR "microbial colonization" OR "early Microbial colonization" OR "dysbiosis" OR "neonatal microbiota") OR ("determinants of neonatal microbiota" OR "mode of delivery" OR "perinatal factors" OR "hospital environment") AND ("Breastfeeding" OR "exclusive breastfeeding" OR "Breast milk microbiota" OR "skin-to-skin contact" OR "Kangaroo care" OR "early skin contact" OR "immediate postpartum contact") AND ("nursing interventions" OR "neonatal nursing care" OR "evidence-based nursing" OR "nursing" OR "nurse"))	2016–2026; English or Spanish; reviews excluded

**Web of Science**	TS=(("newborn" OR "neonate") AND ("Neonatal microbiome" OR "microbial colonization" OR "early Microbial colonization" OR "dysbiosis" OR "neonatal microbiota") OR ("determinants of neonatal microbiota" OR "mode of delivery" OR "perinatal factors" OR "hospital environment") AND ("Breastfeeding" OR "exclusive breastfeeding" OR "Breast milk microbiota" OR "skin-to-skin contact" OR "Kangaroo care" OR "early skin contact" OR "immediate postpartum contact") AND ("nursing interventions" OR "neonatal nursing care" OR "evidence-based nursing" OR "nursing" OR "nurse"))	2016–2026; English or Spanish; reviews excluded
**CINAHL**	TX (("newborn" OR "neonate") AND ("Neonatal microbiome" OR "microbial colonization" OR "early Microbial colonization" OR "dysbiosis" OR "neonatal microbiota") OR ("determinants of neonatal microbiota" OR "mode of delivery" OR "perinatal factors" OR "hospital environment") AND ("Breastfeeding" OR "exclusive breastfeeding" OR "Breast milk microbiota" OR "skin-to-skin contact" OR "Kangaroo care" OR "early skin contact" OR "immediate postpartum contact") AND ("nursing interventions" OR "neonatal nursing care" OR "evidence-based nursing" OR "nursing" OR "nurse"))	2016–2026; English or Spanish; reviews excluded

Table S2. Quality assessment

Criterion	Assessment criterion	Score
P1	Is the study objective clearly defined?	0–2
P2	Is the neonatal population adequately defined and characterized?	0–2
P3	Is the study design appropriate to answer the research question?	0–2
P4	Is the intervention, exposure, or care practice clearly described?	0–2
P5	Are the reported outcomes relevant to the neonatal microbiome or microbiota?	0–2
P6	Are key perinatal factors (e.g., mode of delivery, breastfeeding, or antibiotic exposure) adequately described?	0–2
P7	Are the data collection and analysis methods clearly described?	0–2
P8	Are the reported results consistent with the stated study objective?	0–2
P9	Are the conclusions supported by the reported findings?	0–2
P10	Is the study relevant to the review question?	0–2
P11	Is the overall methodological quality sufficient for inclusion in the evidence synthesis?	0–2

Scoring system: Each criterion was scored as **0 = not fulfilled**, **1 = partially fulfilled**, or **2 = fully fulfilled**, yielding a maximum score of **22 points**.

Studies scoring **18–22 points** were classified as **high quality**, **12–17 points** as **moderate quality**, and **0–11 points** as **low quality**.

Table S3. Methodological quality

Autor	Año	P1	P2	P3	P4	P5	P6	P7	P8	P9	P10	P11	TOTAL	CALIDAD
Akagawa et al. [26]	2019	2	2	2	2	2	2	2	2	2	2	2	22	HIGH☆
Omaña-Covarrubias et al. [25]	2025	2	2	2	1	2	1	2	2	2	1	2	19	HIGH
Farooq et al. [26]	2025	2	1	1	1	2	2	2	2	2	1	1	17	MEDIUM
Seifert et al. [27]	2025	1	1	1	1	2	1	1	2	2	1	1	14	MEDIUM
Mueller et al. [28]	2023	2	2	2	2	2	2	2	2	2	2	2	22	HIGH☆
Matharu et al. [29]	2022	2	2	2	2	2	2	2	2	2	2	2	22	HIGH☆
Raspini et al. [30]	2021	2	2	2	2	2	2	2	2	2	2	2	22	HIGH☆
Wu et al. [31]	2021	2	1	2	1	2	0	2	2	1	1	1	15	MEDIUM
García-Mantrana et al. [32]	2020	2	2	2	1	2	1	2	2	2	1	2	19	HIGH
Zhou et al. [33]	2020	2	2	2	2	2	1	2	2	2	2	2	21	HIGH☆
Nagpal et al. [34]	2017	2	1	2	2	1	2	2	2	2	2	2	20	HIGH
Dominguez-Bello et al. [35]	2016	2	1	2	2	2	1	1	2	2	2	2	19	HIGH
Ajeeb et al. [36]	2024	2	1	2	2	1	2	2	2	2	1	1	18	HIGH
Cabrera-Rubio et al. [37]	2024	2	1	2	2	1	2	2	2	2	1	1	18	HIGH
Klymiuk et al.[38]	2021	2	1	1	0	2	0	2	2	1	0	0	11	LOW

Methodological quality was assessed using eleven predefined criteria: (P1) clarity of the study objective, (P2) adequacy of neonatal population characterization, (P3) appropriateness of the study design, (P4) description of the intervention or exposure, (P5) relevance of microbiome-related outcomes, (P6) reporting of key perinatal factors, (P7) transparency of data collection and analysis methods, (P8) consistency between objectives and results, (P9) validity of the conclusions, (P10) relevance to the review question, and (P11) overall methodological quality.

Table S4. Microbiome assessment and analytical characteristics of the included studies

Author	Biological specimen	Sampling time	Microbiome / laboratory method	Bioinformatics / analytical approach	Main microbiome outcomes	Confounders / covariates considered	Main effect estimates / statistical findings
**Akagawa et al. [23]**	Infant stool	Day 4 and 1 month	16S rRNA gene sequencing	Taxonomic composition and bacterial diversity analyses	Diversity; relative bacterial abundance	Delivery mode; feeding type	At day 4, Bacteroidales and Enterobacteriales were higher after vaginal delivery (p=0.0031; p=0.011), whereas Bacillales and Lactobacillales were higher after cesarean delivery (p=0.012; p=0.0016). Differences were markedly reduced at 1 month.
**Omaña-Covarrubias et al. [25]**	Maternal and neonatal intestinal microbiota	Maternal assessments during pregnancy; neonatal assessment after birth	Intestinal microbiota analysis	Correlation analyses focused on Firmicutes, Bacteroidetes and F/B ratio	Taxonomic composition; F/B ratio; maternal–neonatal correlations	Maternal anthropometric, biochemical and fetal growth variables	Bacteroidetes correlated with birth weight (r=0.2876, p=0.0063), birth length (r=0.5889, p<0.001), and head circumference (r=0.2163, p=0.0418).
**Farooq et al. [26]**	Maternal and infant fecal metagenomes	Birth and months 1, 2, 3, 4, 8 and 12	Shotgun metagenomic analysis of 125 public metagenomes	Metagenomic identification of ARGs, taxa, mobile genetic elements and virulence determinants	Resistome; ARG persistence; microbial carriers; mother–infant transmission	Delivery mode, feeding, gestation and antibiotic exposure examined descriptively	Core ARGs were repeatedly detected in mothers and infants across longitudinal time points; dominant ARG-carrying taxa included Escherichia coli, Klebsiella and Streptococcus.
**Seifert et al. [27]**	Maternal vaginal swab; newborn anterior-neck skin and oral swabs	Immediately after delivery	16S rRNA amplicon sequencing; Illumina MiSeq; skin V1–V3 and oral/vaginal V3–V4 regions	CosmosID; Chao1; relative abundance; Wilcoxon rank-sum; Spearman correlations	Alpha diversity; relative abundance; maternal–newborn microbial correlations	Pre-pregnancy BMI; delivery mode	Peptoniphilus was higher in newborn skin of mothers with obesity (p=0.007); oral Ureaplasma was higher after cesarean delivery (p=0.046).

Author	Biological specimen	Sampling time	Microbiome / laboratory method	Bioinformatics / analytical approach	Main microbiome outcomes	Confounders / covariates considered	Main effect estimates / statistical findings
**Mueller et al. [28]**	Maternal vaginal samples; neonatal skin and stool	Immediately after birth; day 1; transitional stool; day 30	16S rRNA sequencing and bacterial-load assessment	Shannon diversity; beta diversity; ANCOM; maternal source tracking	Alpha/beta diversity; ASVs; maternal engraftment	Randomization; placebo control; baseline characteristics	Seeding reduced Shannon diversity in skin at day 1 (p=0.047), transitional stool (p=0.02), and stool at day 30 (p=0.01). Treatment explained 11.5% of day-1 skin beta-diversity variance (p=0.02).
**Matharu et al. [29]**	Maternal and infant fecal samples	Infant: 3 weeks, 3, 6 and 12 months; maternal: ≤2 weeks before delivery	Shotgun metagenomic sequencing	Taxonomic/functional profiling and hierarchical clustering	Taxonomic and functional maturation; developmental trajectories; alpha/beta diversity	Delivery mode; intrapartum antibiotics; feeding and other early-life exposures	Three longitudinal developmental trajectories were identified; trajectories were strongly associated with mode of delivery and Bacteroides abundance.
**Raspini et al. [30]**	Infant meconium and fecal samples	Birth (T0) and 6 months (T2)	16S rRNA V3–V4 amplicon sequencing	Shannon index; OTUs; PCA; clustering/DAPC; multivariate analyses	Alpha/beta diversity; relative abundance; community structure	Maternal BMI, gestational weight gain, delivery, feeding, weaning, siblings	Exclusive breastfeeding was associated with lower Shannon diversity at 6 months (p=0.018); no single factor independently explained overall microbiota development.
**Wu et al. [31]**	Maternal oral cavity, amniotic fluid, placenta, neonatal oral cavity	At cesarean delivery	16S rRNA high-throughput sequencing; Illumina MiSeq	OTU analysis and PCoA	Shared OTUs; community similarity; taxonomic composition	Homogeneous cohort: healthy term pregnancies, cesarean delivery, no recent antimicrobials	Up to 635 shared OTUs were identified; Streptococcus, Fusobacterium and Prevotella were prominent shared genera; community structures showed similarities across the four sampling sites.
**García-Mantrana et al. [32]**	Maternal and neonatal fecal samples	At birth; infant clinical follow-up to 18 months	16S rRNA sequencing, Illumina	Maternal microbial clustering; taxonomic analyses; longitudinal growth analyses	Maternal/neonatal composition; microbial clusters; infant growth trajectories	Maternal diet, antibiotics, BMI/weight gain, delivery mode, feeding	A Prevotella-dominated and a Ruminococcus-dominated maternal cluster were identified. Higher fiber, omega-3 and polyphenol intake characterized the latter; maternal clusters were

Author	Biological specimen	Sampling time	Microbiome / laboratory method	Bioinformatics / analytical approach	Main microbiome outcomes	Confounders / covariates considered	Main effect estimates / statistical findings
							associated with neonatal microbiota and growth according to delivery mode.
**Zhou et al. [33]**	Maternal vaginal swab and neonatal meconium	At delivery	16S rRNA gene sequencing	OTU analysis; Bray–Curtis dissimilarity; PERMANOVA	Microbial composition; Lactobacillus abundance; mother–infant dissimilarity	Gestational age; perinatal antibiotic exposure	Maternal vaginal Lactobacillus was markedly lower with antibiotic exposure; e.g., full-term unexposed 66.17% vs exposed 12.86% ($p<0.001$). Full-term neonatal meconium also contained more Lactobacillus than preterm meconium ($p=0.007$).
**Nagpal et al. [34]**	Infant feces	7 days; 1, 3 and 6 months; 3 years	qPCR for toxigenic C. perfringens; RT-qPCR for other gut bacteria	Longitudinal quantitative bacterial comparisons	C. perfringens carriage; bacterial counts; organic acids	Delivery mode; feeding type	Cesarean-born infants showed higher C. perfringens carriage and lower B. fragilis group, bifidobacteria and lactobacilli. Lactobacillus prevalence at 3 months was 31% in cesarean vs 65% in vaginally delivered infants ($p=0.03$).
**Dominguez-Bello et al. [35]**	Maternal vaginal samples; infant anal, oral and skin samples	Birth and repeated sampling during first 30 days	16S rRNA sequencing	Unweighted UniFrac; PCoA; SourceTracker; phylogenetic diversity	Community similarity; diversity; maternal microbial transfer	Delivery mode; exposure versus no exposure to maternal vaginal fluids	Distances between vaginally delivered and exposed cesarean infants were significantly smaller than between vaginally delivered and unexposed cesarean infants ($p<0.01$).
**Ajeeb et al. [36]**	Human milk	Early lactation 6–46 days; late lactation 109–184 days	16S rRNA gene sequencing	ANCHOR pipeline; DESeq2; cluster/correlation analyses	Milk microbiota composition and differential abundance	Maternal macro/micronutrient intake; lactation stage; infant growth status	Multiple positive and negative associations were observed between maternal nutrient intake and milk microbial taxa; differentially abundant taxa were

Author	Biological specimen	Sampling time	Microbiome / laboratory method	Bioinformatics / analytical approach	Main microbiome outcomes	Confounders / covariates considered	Main effect estimates / statistical findings
							also associated with infant growth z-scores.
**Cabrera-Rubio et al. [37]**	Infant fecal samples	1 month of age	16S rRNA sequencing, Illumina MiSeq	Alpha/beta diversity analyses; Bray–Curtis; PCoA; multivariable analyses	Diversity; microbial composition; taxa associated with maternal diet	Maternal diet index; maternal and infant characteristics included in adjusted analyses	Maternal diet-index groups showed differences in infant microbial community structure and specific taxa; associations remained after consideration of relevant maternal and perinatal variables.
